# Data on health risk assessment of fluoride in drinking water in the Khash city of Sistan and Baluchistan province, Iran

**DOI:** 10.1016/j.dib.2018.08.139

**Published:** 2018-08-31

**Authors:** Abooalfazl Azhdarpoor, Majid Radfard, Massuomeh Rahmatinia, Hassan Hashemi, Bayram Hashemzadeh, Samira Nabavi, Hamed Akbari, Hesam Akbari, Amir Adibzadeh

**Affiliations:** a Department of Environmental Health Engineering, School of Public Health, Shiraz University of Medical Sciences, Shiraz, Iran; b Student Research Committee, School of Public Health, Shahid Beheshti University of Medical Sciences, Tehran, Iran.; c Khoy University of Medical Sciences, Khoy, Iran; d Department of Environmental Health Engineering, School of Public Health, Tehran, Tehran, Iran; e Health Research Center, Lifestyle, Institute, Baqiyatallah University of Medical Sciences, Tehran

**Keywords:** Drinking water, Fluoride, Risk assessment, Khash, Iran

## Abstract

According to studies, high concentration of fluoride in drinking water has adverse health effects such as dental and skeletal fluorosis. This data analyzes the concentrations and health risks of fluoride in 30 drinking water samples collected from 11 villages of the Khash city, Sistan and Baluchistan province in Iran. Fluoride concentration was measured using SPADNS method according to the standard method for examination of water and wastewater. Data indicated that average fluoride concentration in drinking water was 0.731 mg L^−1^. The mean estimated daily intake (EDI) values for fluoride in different groups of infants, children, teenagers and adults were 0.0058, 0.0414, 0.0292 and 0.0234 mg/kg, respectively. Also, risk assessment data indicated that hazard quotient (HQ) value of groundwater samples was less than one in 90% of samples in age groups of infants, children, teenagers and adults.

**Specifications Table**

**Table:** 

Subject area	Water quality and risk assessment
More specific subject area	Water fluoride
Type of data	Table and Figure
How data was acquired	All water samples were analyzed using SPADNS according to standard method for method examination water and wastewater. Also, fluoride concentration was determined by Spectrophotometer (DR/5000, USA).
Data format	Raw, Analyzed
Experimental factors	Water samples were stored in polyethylene bottles in a dark place at room temperature until analysis.
Experimental features	Determine the concentration levels of fluoride
Data source location	Khash region, of Sistan and Baluchistan province, Iran
Data accessibility	The data are available with this article

**Value of the data**

• Continuous monitoring of water resources for fluoride is essential due to prevention of undesirable health effects.
• The data of this research showed that the fluoride concentration in all of water samples was less than the maximum permissible limits (1.5 mg/L) according to world health organization (WHO) guidelines [1].
• Based on the data, the amount of fluoride in some sampling points was less than 0.5 mg/L, so it is recommended to use toothpaste containing fluoride.
• Health risk assessment and data analysis indicated that HQ value was > 1 for the age group of children in three sampling areas, so select of a suitable resource of drinking water for this age group could be recommended.

## 1 Data

**Fig. 1:**
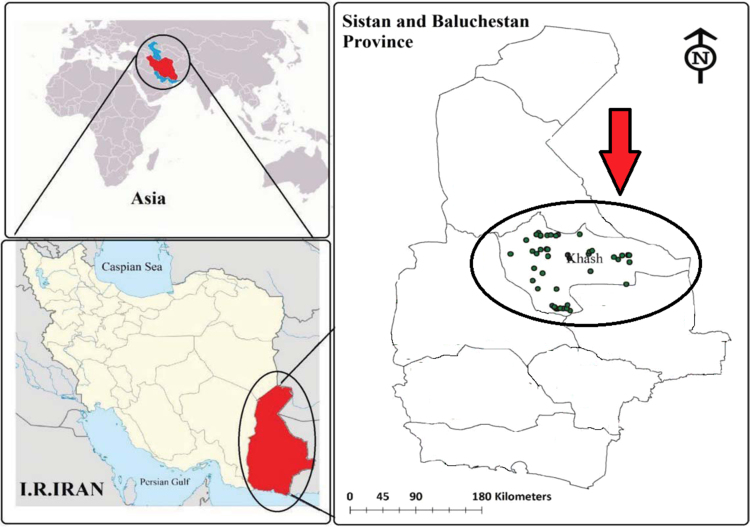
Location of nitrate sampling in drinking water in the Khash area.

**Fig. 2:**
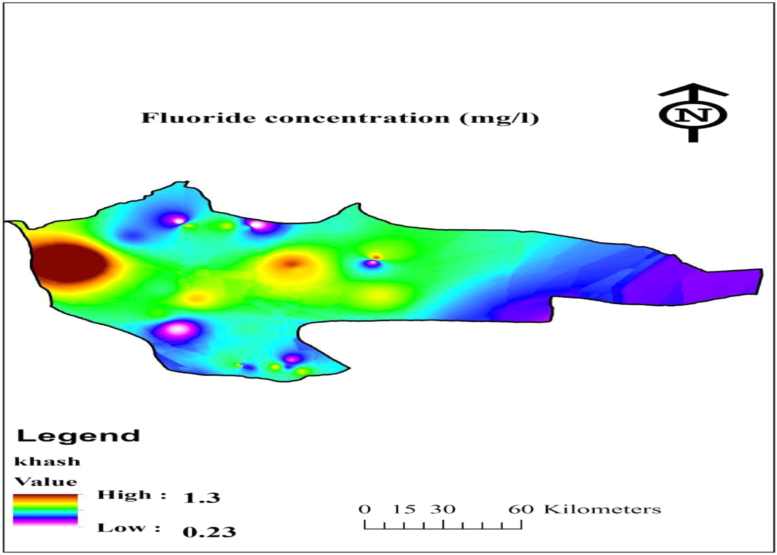
Geological distribution of fluoride in Khash area.

Table 1 shows the parameters used to calculate the fluoride risk assessment in water samples. Fluoride concentration and EDI and hazard quotient HQ for the four populations of water consumers in the data have been shown in Table 2. Also, geological distribution of fluoride in the research area is shown in Fig. 1, Fig. 2.

**Table 1:** Parameters used to in the present data for the fluoride risk assessment in water drinking [1], [2], [3], [4], [5], [6], [7].

Parameter	Risk exposure factors	Values for groups				Unit
		Infants	Children	Teenagers	Adults	
Fluoride	*C_f_*	–	–	–	–	mg/L
	*C_d_*	0.08	0.85	2	2.5	Liter/day
	*B_w_*	10	15	50	78	kg
	RfD	0.06	0.06	0.06	0.06	mg/kg day

**Table 2:** Fluoride concentration and EDI and HQ for the four populations of water consumers.

Nos.	Fluoride	EDI				HQ			
	concentration	Infants	Children	Teenagers	Adults	Infants	Children	Teenagers	Adults
1	0.910	0.0073	0.0516	0.0364	0.0292	0.1213	0.8594	0.6067	0.4861
2	0.960	0.0077	0.0544	0.0384	0.0308	0.1280	0.9067	0.6400	0.5128
3	0.480	0.0038	0.0272	0.0192	0.0154	0.0640	0.4533	0.3200	0.2564
4	1.050	0.0084	0.0595	0.0420	0.0337	0.1400	0.9917	0.7000	0.5609
5	0.750	0.0060	0.0425	0.0300	0.0240	0.1000	0.7083	0.5000	0.4006
6	0.540	0.0043	0.0306	0.0216	0.0173	0.0720	0.5100	0.3600	0.2885
7	1.360	0.0109	0.0771	0.0544	0.0436	0.1813	1.2844	0.9067	0.7265
8	0.340	0.0027	0.0193	0.0136	0.0109	0.0453	0.3211	0.2267	0.1816
9	0.940	0.0075	0.0533	0.0376	0.0301	0.1253	0.8878	0.6267	0.5021
10	0.820	0.0066	0.0465	0.0328	0.0263	0.1093	0.7744	0.5467	0.4380
11	0.980	0.0078	0.0555	0.0392	0.0314	0.1307	0.9256	0.6533	0.5235
12	0.720	0.0058	0.0408	0.0288	0.0231	0.0960	0.6800	0.4800	0.3846
13	0.490	0.0039	0.0278	0.0196	0.0157	0.0653	0.4628	0.3267	0.2618
14	0.650	0.0052	0.0368	0.0260	0.0208	0.0867	0.6139	0.4333	0.3472
15	0.390	0.0031	0.0221	0.0156	0.0125	0.0520	0.3683	0.2600	0.2083
16	0.700	0.0056	0.0397	0.0280	0.0224	0.0933	0.6611	0.4667	0.3739
17	0.620	0.0050	0.0351	0.0248	0.0199	0.0827	0.5856	0.4133	0.3312
18	0.230	0.0018	0.0130	0.0092	0.0074	0.0307	0.2172	0.1533	0.1229
19	0.920	0.0074	0.0521	0.0368	0.0295	0.1227	0.8689	0.6133	0.4915
20	0.720	0.0058	0.0408	0.0288	0.0231	0.0960	0.6800	0.4800	0.3846
21	1.070	0.0086	0.0606	0.0428	0.0343	0.1427	1.0106	0.7133	0.5716
22	0.240	0.0019	0.0136	0.0096	0.0077	0.0320	0.2267	0.1600	0.1282
23	0.780	0.0062	0.0442	0.0312	0.0250	0.1040	0.7367	0.5200	0.4167
24	0.820	0.0066	0.0465	0.0328	0.0263	0.1093	0.7744	0.5467	0.4380
25	1.110	0.0089	0.0629	0.0444	0.0356	0.1480	1.0483	0.7400	0.5929
26	0.570	0.0046	0.0323	0.0228	0.0183	0.0760	0.5383	0.3800	0.3045
27	0.740	0.0059	0.0419	0.0296	0.0237	0.0987	0.6989	0.4933	0.3953
28	0.740	0.0059	0.0419	0.0296	0.0237	0.0987	0.6989	0.4933	0.3953
29	0.430	0.0034	0.0244	0.0172	0.0138	0.0573	0.4061	0.2867	0.2297
30	0.860	0.0069	0.0487	0.0344	0.0276	0.1147	0.8122	0.5733	0.4594
Mean	0.731	0.0058	0.0414	0.0292	0.0234	0.0975	0.6904	0.4873	0.3905
Max	1.360	0.0109	0.0771	0.0544	0.0436	0.1813	1.2844	0.9067	0.7265
Min	0.230	0.0018	0.0130	0.0092	0.0074	0.0307	0.2172	0.1533	0.1229
SD	0.267	0.0021	0.0151	0.0107	0.0086	0.0356	0.2525	0.1782	0.1428
1053IR standard	0.7–1.2(mg/L)								
WHO guideline	0.5–1.5(mg/L)

## 2 Experimental design, materials and methods

### 2.1 Description of study area

Khash city is located in Sistan and Baluchistan Province of Iran between the latitudes 28° 13′ N and Longitudes 61° 13′ E. According to the demographic information of Iran, this city is populated with almost 173,821 with an area 19.376 square kilometers. Khah city has a warm and dry climate and the highest and lowest air temperatures are 37 °C and − 7 °C, respectively [8], [9], [10], [11].

### 2.2 Determination of fluoride concentration in drinking water

The samples of this data were collected from rural drinking water sources from 11 villages of the Khash city from 2016 to 2017. 30 samples were taken from the ground water wells in sterile polyethylene bottles and then transported to laboratory. Fluoride concentration of water samples were analyzed by SPADNS method at wavelength of 580 nm and measured using a Spectrophotometer (DR/5000, USA) [12], [13], [14], [15], [16], [17], [18]. Excel software has been used for analysis of data. Finally, fluoride concentration was compared with WHO guidelines [18], [19], [20], [21], [22].

### 2.3 Risk assessment of fluoride

Human health risk assessment is defined as the probability of adverse health effects to humans that may be exposed to chemicals in an infected environment [1], [2]. So, the health risk of fluoride through consumption of drinking water was assessed in rural population of Khash city. After the collection and analysis of samples divided the population into four age groups according to a study by Mahmood Yousefi et al. as follow [2]: infants (less than 2 years old), children (2 to < 6 years old), teenagers (6 to < 16 years old) and adults (≥ 16 years old). The daily exposure to fluoride was calculated in these groups using Eq. (1):

(1) $$EDI=\frac{C_{f}\times C_{d}}{B_{w}}$$

where EDI is estimation of daily fluoride consumption (mg/kg day), *C_f_* is fluoride concentration in drinking water (mg/L), *C_d_* is average daily drinking water intake and *B_w_* is body weight (kg). Water consumption and body weight data were measured based on a questionnaire that was asked from target groups (infants, children, adolescents and adults). The average water consumption rates in infants (0–2 years old), children (2–6 years old), teenagers (6–16 years old) and adults (≥ 16 years old) were 0.08, 0.85, 2 and 2.5 L day^−1^, respectively. Body weight of target groups were considered 10, 15, 50 and 78 kg, respectively. HQ is the non-carcinogenic risk of fluoride to human health that was calculated using Eq. (2) [1], [2], [3], [4], [5].

(2) $$HQ=\frac{EDI}{RfD}$$

The reference dose (RfD, mg/kg d) was used to estimate of the daily exposure of fluoride in the community that is likely to be without a considerable risk of adverse effects during a lifetime. The oral reference doses of fluoride is 0.06 mg kg^−1^d^−1^ according to the integrated Risk Information System, USEPA. A value of HQ less than one indicates a negligible risk of non-carcinogenic effects and HQ higher than one indicates a significant risk level.
